# New syntheses of 5,6- and 7,8-diaminoquinolines

**DOI:** 10.3762/bjoc.9.302

**Published:** 2013-11-27

**Authors:** Maroš Bella, Viktor Milata

**Affiliations:** 1 Institute of Chemistry, Slovak Academy of Sciences, Dúbravská cesta 9, SK-845 38 Bratislava, Slovakia; 2 Department of Organic Chemistry, Faculty of Chemical and Food Technology, Slovak University of Technology, Radlinského 9, SK-812 37 Bratislava, Slovakia

**Keywords:** chlorination, deselenation, nitrogen heterocycles, *o*-diaminoquinolines, selenadiazoloquinolones

## Abstract

The synthesis of 5,6- and 7,8-diaminoquinoline derivatives starting from angularly annelated selenadiazoloquinolones is presented. Simple chlorination of the pyridone ring followed by reductive deselenation of the 1,2,5-selenadiazole ring afforded novel 4-chloro-*o*-diaminoquinolines. Dechlorination of 4-chloro-7,8-diaminoquinoline gave 7,8-diaminoquinoline hydrochloride which was successfully employed as starting material in the synthesis of condensed nitrogen heterocycles.

## Introduction

*o*-Diaminoquinolines, in particular 5,6- and 7,8-diaminoquinolines, represent valuable intermediates in the synthesis of nitrogen-containing heterocycles [1–3] including food-borne carcinogens [4].

To date, the known procedures for the synthesis of 7,8-diaminoquinoline have started from 3-nitroaniline or 3-chloroaniline. Initially, 7,8-diaminoquinoline was prepared by Renshaw et al. [5] by coupling 7-aminoquinoline with benzenediazonium chloride followed by the reduction of the resulting azo-dye with SnCl_2_·2H_2_O. The second method relies on the Skraup reaction [6] of 3-nitroaniline affording 7-nitroquinoline in a low yield (14%). The latter was reduced with iron in acetic acid to 7-aminoquinoline which was subsequently tosylated, nitrated in position 8 and detosylated to yield 7-amino-8-nitroquinoline. In the final step, the reduction of aminonitroquinoline with SnCl_2_·2H_2_O provided 7,8-diaminoquinoline [7]. Another approach is based on the amination of 7-nitroquinoline in position 8 with hydroxylamine under basic conditions. The resulting 8-amino-7-nitroquinoline was reduced with SnCl_2_·2H_2_O [8] or hydrazine hydrate on Raney nickel as a catalyst [9] to obtain 7,8-diaminoquinoline. In the last method, the Skraup reaction of 3-chloroaniline led to 7-chloroquinoline which, after nitration, replacement of the chlorine atom by the amino group and catalytic hydrogenation on 5% palladium on charcoal, yielded 7,8-diaminoquinoline [10].

The preparation of 5,6-diaminoquinoline is more effective because the Skraup reaction of 4-nitroaniline produces 6-nitroquinoline as a sole product in moderate yield (47%). The latter, on treatment with hydroxylamine hydrochloride in the presence of KOH [11] followed by the reduction with hydrazine hydrate on Raney nickel, provided 5,6-diaminoquinoline in almost quantitative yield [12].

A disadvantage of the syntheses of *o*-diaminoquinolines described above is that they require the Skraup reaction, which usually gives low yields and can be violently exothermic [6]. Moreover, in the case of 3-nitro- and 3-chloroaniline, the Skraup reaction affords a mixture of 5- and 7-substituted quinolines, making their separation necessary. To avoid these drawbacks, the readily available angularly annelated selenadiazoloquinolones, prepared as detailed in our previous papers [13–14], were employed as the precursors of *o*-diaminoquinoline derivatives. The preparation and application of 7,8-diaminoquinoline hydrochloride in the synthesis of nitrogen heterocycles is discussed in datail.

## Results and Discussion

**Scheme 1:**
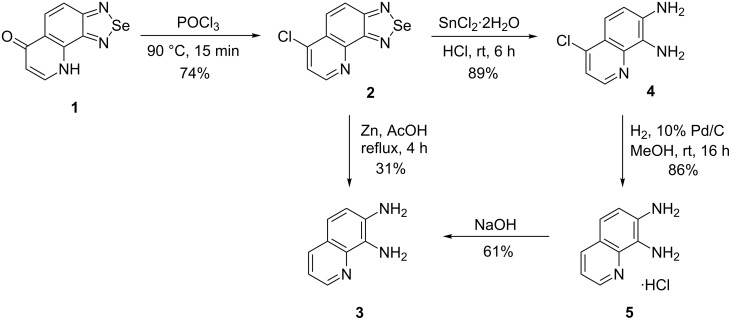
Synthesis of 7,8-diaminoquinoline hydrochloride (**5**).

In the present approach, selenadiazoloquinolones **1** and **9** represent a masked form of the target *o*-diaminoquinolines where the *o*-phenylenediamine moiety is protected as the 2,1,3-benzoselenadiazole skeleton while the pyridine ring can be obtained by transformation of the pyridone core [15]. The synthesis of 7,8-diaminoquinoline (**3**) starting from selenadiazolo[3,4-*h*]quinolone **1** is depicted in Scheme 1. In the first step, the aromatization of the pyridone ring by chlorination with POCl_3_ in DMF, which worked reliably on related azoloquinolones [15], was attempted. However, in the case of selenadiazolo[3,4-*h*]quinolone **1,** only decomposition products were observed. On the other hand, chlorination with neat POCl_3_ at 90 °C afforded 6-chloroselenadiazoloquinoline **2** in 74% yield after recrystallisation from toluene (Scheme 1). The temperature of the chlorination (90 °C) was found to be crucial, since heating of the reaction mixture under reflux led to a rapid decomposition. The catalytic hydrogenation of 6-chloroderivative **2** using 10% palladium on charcoal or Raney nickel did not afford 7,8-diaminoquinoline (**3**) in a single step. Finally, the simultaneous deselenation and dechlorination of 6-chloroderivative **2** with zinc in refluxing acetic acid accessed 7,8-diaminoquinoline (**3**) in 31% yield. Due to the low yield of diaminoquinoline **3** following the treatment with zinc in acetic acid, the reduction of 6-chloroderivative **2** was performed in two successive steps. First, the reductive deselenation with SnCl_2_·2H_2_O in concentrated hydrochloric acid afforded 4-chlorodiaminoquinoline **4** in 89% yield. The subsequent catalytic hydrogenation on 10% palladium on charcoal in the absence of base, after filtration of the catalyst and evaporation of methanol, gave 7,8-diaminoquinoline hydrochloride (**5**) in high yield (Scheme 1). It was notable that the use of sodium hydroxide as a base during the catalytic hydrogenation either on 10% palladium on charcoal or Raney nickel did not lead to diaminoquinoline **3**. After alkalisation of the hydrochloride **5** with a sodium hydroxide solution, diaminoquinoline **3** was isolated in good yield (61%). However, 7,8-diaminoquinoline (**3**) is relatively unstable and decomposes slowly both during isolation or subsequent to it. Hence, the isolation of free diaminoquinoline **3** is practically irreproducible due to this instability. Accordingly, it is far more convenient to employ hydrochloride **5** as the starting material in the subsequent reactions, whether isolated or prepared in situ.

**Scheme 2:**
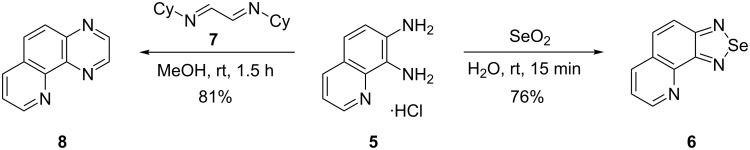
Application of hydrochloride **5** in the syntheses of nitrogen heterocycles.

Next, hydrochloride **5** was applied as the substrate in the synthesis of nitrogen heterocycles (Scheme 2). The treatment of hydrochloride **5** with selenium dioxide in water at room temperature afforded selenadiazoloquinoline **6** in 76% yield (Scheme 2) in comparison with 18% yield obtained by heating of diaminoquinoline **3** with selenium dioxide in dioxane under reflux [9]. To date, pyridoquinoxaline **8** has been prepared in a poor yield by application of the Skraup synthesis to 5-acetylamidoquinoxaline [16]. Notably, 7,8-diaminoquinoline dihydrochloride condensed with glyoxal bisulfite only with the amino group in position 7 and the pyrazine ring was not closed [17]. In the present case, the cyclocondensation of hydrochloride **5** with diimine **7** [18–19] proceeded smoothly at room temperature affording pyridoquinoxaline **8** in high yield (Scheme 2).

**Scheme 3:**
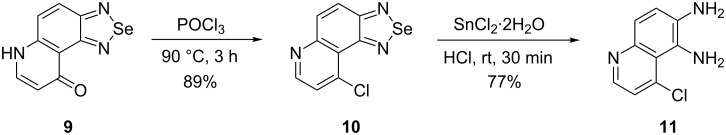
Synthesis of 4-chloro-5,6-diaminoquinoline (**11**).

The same reaction sequence (chlorination and reductive deselenation) was applied to selenadiazolo[3,4-*f*]quinolone **9** (Scheme 3). The treatment of selenadiazoloquinolone **9** with POCl_3_ led to 9-chloroselenadiazoloquinoline **10** in high yield. Unlike the reduction of 6-chloroderivative **2**, the reductive deselenation of 9-chloroderivative **10** proceeded much more rapidly and 4-chloro-5,6-diaminoquinoline (**11**) was isolated in 77% yield. However, it should be noted that this reductive deselenation required careful monitoring by TLC (CHCl_3_/MeOH 100:1, *R*_f_ = 0.10) at 10 min intervals in order to detect its accurate termination, because prolongation of the reaction time results in decreased yields.

## Conclusion

Angularly annelated selenadiazoloquinolone **1** and **9** were successfully employed as starting materials in the synthesis of 5,6- and 7,8-diaminoquinoline derivatives. 7,8-Diaminoquinoline hydrochloride (**5**) was prepared in three steps in 57% overall yield starting from selenadiazolo[3,4-*h*]quinolone **1**. Hydrochloride **5** was applied as an important intermediate in the synthesis of nitrogen heterocycles. Hydrochloride **5** affords the advantages of stability in air and simple isolation; it could also be used in further reactions prepared in situ without isolation. In addition, 4-chloro-*o*-diaminoquinolines **4** and **11** also represent valuable substrates for the preparation of chlorinated nitrogen heterocycles.

## Experimental

**General.** Thin-layer chromatography (TLC) was performed on aluminium plates precoated with 0.2 mm silica gel (25 μm) containing fluorescent indicator 254 nm (Fluka) and stains were visualised by UV light (254 nm or 366 nm). Flash liquid chromatography (FLC) was performed on silica gel [Normasil 60 (43–60 μm)]. Melting points were measured on a Koffler block and are uncorrected. ^1^H NMR and ^13^C NMR spectra were recorded on a Varian Mercury 300 MHz spectrometer at 25 °C. The operating frequencies were 300 MHz for ^1^H and 75.5 MHz for ^13^C nuclei. Chemical shifts (δ) are reported in ppm and coupling constants (*J*) are given in Hz. Elemental analyses were determined using a Thermo Finnigan Flash EA 1112 instrument.

**6-Chloro-[1,2,5]selenadiazolo[3,4-*h*]quinoline (2).** A mixture of selenadiazolo[3,4-*h*]quinolone **1** (5.0 g, 20.0 mmol) and POCl_3_ (10 mL, 16.4 g, 0.1 mol) was stirred at 90 °C for 15 min. After the reaction was complete, the mixture was cooled to 0 °C in an ice bath followed by the addition of crushed ice (~45 g) in one portion under stirring. Once the ice was melted, the resulting brown solution was alkalised with a 20% NaOH solution under cooling in the ice bath. The brown precipitate was collected by suction, washed with acetone and dried. Recrystallisation from toluene gave 6-chloroselenadiazoloquinoline **2** (4.0 g, 74%) as golden plates; mp 237–240 °C; ^1^H NMR (300 MHz, TFA-*d*) δ 8.08 (d, *J* = 9.8 Hz, 1H, H-4), 8.17 (d, *J* = 6.0 Hz, 1H, H-7), 8.30 (d, *J* = 9.8 Hz, 1H, H-5), 8.86 (d, *J* = 6.0 Hz, 1H, H-8); ^13^C NMR (75 MHz, TFA-*d*) δ 127.1, 128.5, 128.8, 131.1, 136.8, 143.6, 151.9, 158.5, 161.4; anal. calcd for C_9_H_4_ClN_3_Se: C, 40.25; H, 1.50; N, 15.65; found: C, 40.19; H, 1.48; N, 15.70.

**4-Chloro-7,8-diaminoquinoline (4).** SnCl_2_·2H_2_O (4.0 g, 17.7 mmol) was added in small portions to a stirred suspension of 6-chloroselenadiazoloquinoline **2** (1.0 g, 3.7 mmol) in concentrated HCl (33 mL) at room temperature and stirring was continued for 6 h. Next, the reaction mixture was diluted with water (30 mL), the insoluble material was removed by filtration under reduced pressure and the filter cake was washed thoroughly with water (150–200 mL). The filtrate was subsequently alkalised with a 20% NaOH solution under cooling in an ice bath and the resulting yellow suspension was extracted with ethyl acetate (3 × 100 mL). The combined organic phases were dried with Na_2_SO_4_, filtered and the solvent evaporated under reduced pressure to afford 4-chlorodiaminoquinoline **4** (0.64 g, 89%) as a yellow solid which was used in the next reaction without further purification; mp 136–139 °C. ^1^H NMR (300 MHz, DMSO-*d*_6_) δ 5.19 (br s, 2H, NH_2_), 5.25 (br s, 2H, NH_2_), 7.14 (d, *J* = 8.7 Hz, 1H, H-6), 7.28 (d, *J* = 8.7 Hz, 1H, H-5), 7.29 (d, *J* = 4.6 Hz, 1H, H-3), 8.50 (d, *J* = 4.6, 1H, H-2); ^13^C NMR (75 MHz, DMSO-*d*_6_) δ 110.9, 116.7, 118.5, 119.4, 127.0, 133.7, 138.3, 140.7, 146.8; anal. calcd for C_9_H_8_ClN_3_: C, 55.83; H, 4.16; N, 21.70; found: C, 55.92; H, 4.20; N, 21.61.

**7,8-Diaminoquinoline hydrochloride (5).** 10% Pd/C (0.40 g) was added to a solution of 4-chlorodiaminoquinoline **4** (0.64 g, 3.3 mmol) in methanol (35 mL) and the resulting mixture was stirred under H_2_ atmosphere (balloon) overnight at room temperature. Once the reaction was complete, the catalyst was removed by filtration and the dark red filtrate was evaporated under reduced pressure. CHCl_3_ (35 mL) was added to the dark violet solid thus obtained and the resulting suspension was stirred for 15 min. Insoluble material was collected by suction, washed with CHCl_3_ and dried to give hydrochloride **5** (0.55 g, 86%) as a dark violet solid which was used in the next reactions without further purification; mp >230 °C (dec.). ^1^H NMR (300 MHz, DMSO-*d*_6_) δ 7.31 (d, 1H, *J* = 8.8 Hz, H-6), 7.41 (dd, 1H, *J* = 8.0, 5.0 Hz, H-3), 7.53 (d, 1H, *J* = 8.8 Hz, H-5), 8.48 (dd, 1H, *J* = 8.0, 1.1 Hz, H-4), 8.75 (dd, 1H, *J* = 5.0, 1.5 Hz, H-2); ^13^C NMR (75 MHz, DMSO-*d*_6_) δ 116.7, 120.7, 121.3, 122.8, 140.3, 145.2, 164.2 (not all ^13^C carbon signals were observed); anal. calcd for C_9_H_10_ClN_3_: C, 55.25; H, 5.15; N, 21.48, found: C, 55.34; H, 5.18; N, 21.55.

**7,8-Diaminoquinoline (3). ***Method A* (alkalisation of hydrochloride **5**). A solution of hydrochloride **5** (0.24 g, 1.2 mmol) in water (25 mL) was alkalised with a few drops of 20% NaOH solution. The resulting solution was filtered under reduced pressure and the filtrate was extracted with ethyl acetate (4 × 15 mL). The combined organic phases were dried with Na_2_SO_4_, filtered and evaporated under reduced pressure as rapidly as possible to afford diaminoquinoline **3** (0.12 g, 61%) as a dark grey solid which was characterised without further purification; mp 100–102 °C (Ref. [8] mp 102 °C). ^1^H NMR (300 MHz, DMSO-*d*_6_) δ 5.07 (br s, 4H, 2×NH_2_), 7.02 (d, *J* = 8.6 Hz, 1H, H-6), 7.05 (d, *J* = 8.6 Hz, 1H, H-5), 7.11 (dd, *J* = 8.1, 4.2 Hz, 1H, H-3), 8.00 (dd, *J* = 8.1, 1.7 Hz, 1H, H-4), 8.59 (dd, *J* = 4.2, 1.7 Hz, 1H, H-2); ^13^C NMR (75 MHz, DMSO-*d*_6_) δ 115.5, 116.7, 118.7, 121.4, 126.3, 132.6, 135.5, 137.9, 147.3; anal. calcd for C_9_H_9_N_3_: C, 67.90; H, 5.70; N, 26.40; found: C, 68.00; H, 5.65; N, 26.47.

*Method B* (reduction of 6-chloroselenadiazoloquinoline **2**). Powdered Zn (0.58 g, 8.93 mmol) was added to a stirred suspension of 6-chloroselenadiazoloquinoline **2** (0.20 g, 0.74 mmol) in acetic acid and the mixture was heated under reflux for 4 h. After the reaction was complete, the hot mixture was filtered, the filter cake was washed with acetic acid (15 mL) and the filtrate was evaporated under reduced pressure. The resulting orange oily residue was dissolved in water (7 mL) and alkalised with a 10% NaOH solution. The yellow suspension was extracted with ethyl acetate (4 × 15 mL). The combined organic phases were dried with Na_2_SO_4_, filtered and evaporated under reduced pressure as rapidly as possible to afford diaminoquinoline **3** (37 mg, 31%) as a dark grey solid which was characterised without further purification; mp 99–101 °C (Ref. [8] mp 102 °C). The ^1^H and ^13^C NMR spectral data were in accordance with those of diaminoquinoline **3** prepared by the alkalisation of hydrochloride **5**.

**[1,2,5]Selenadiazolo[3,4-*h*]quinoline (6).** SeO_2_ (58.6 mg, 0.53 mmol) dissolved in water (1 mL) was added to a stirred solution of hydrochloride **5** (100 mg, 0.51 mmol) in water (1 mL) and stirring was continued for an additional 15 min at room temperature. Next, the reaction mixture was alkalised with a few drops of 30% NaOH solution and the resulting grey precipitate was collected by suction, washed with water (15 mL) and dried. The mother liquor was extracted with CHCl_3_ (1 × 20 mL), the organic layer was dried with Na_2_SO_4_, filtered and evaporated under reduced pressure. The combined solids obtained after separation by suction and extraction were purified by FLC (silica gel, CHCl_3_/MeOH 100:1, *R*_f_ = 0.31) to give selenadiazoloquinoline **6** (92 mg, 76%) as brownish needles; mp 153–155 °C (Ref. [9] mp 150–151 °C). ^1^H NMR (300 MHz, CDCl_3_) δ 7.62 (dd, 1H, *J* = 8.0, 4.4 Hz, H-7), 7.71 (d, 1H, *J* = 9.5 Hz, H-4), 7.77 (d, 1H, *J* = 9.5 Hz, H-5), 8.14 (dd, 1H, *J* = 8.0, 1.4 Hz, H-6), 9.04 (dd, 1H, *J* = 4.4, 1.4 Hz, H-8); ^13^C NMR (75 MHz, CDCl_3_) δ 123.1, 124.0, 128.6, 130.6, 136.0, 144.4, 150.1, 158.5, 160.7; anal. calcd for C_9_H_5_N_3_Se: C, 46.17; H, 2.15; N, 17.95, found: C, 46.20; H, 2.14; N, 18.00.

**Pyrido[2,3-*f*]quinoxaline (8).** Diimine **7** (113 mg, 0.51 mmol) was added to a stirred solution of hydrochloride **5** (100 mg, 0.51 mmol) in MeOH (8 mL) and stirring was continued at room temperature for 1.5 h. The volatiles were evaporated under reduced pressure and the residue was purified by FLC (silica gel, CHCl_3_/MeOH 100:1, *R*_f_ = 0.25) to afford pyridoquinoxaline **8** (75 mg, 81%) as an off-white solid; mp 147–149 °C (Ref. [16] mp 146.5–147.5 °C). ^1^H NMR (300 MHz, CDCl_3_) δ 7.69 (dd, 1H, *J* = 8.1, 4.3 Hz, H-8), 8.03 (d, 1H, *J* = 9.1 Hz, H-5), 8.08 (d, 1H, *J* = 9.1 Hz, H-6), 8.30 (dd, 1H, *J* = 8.1, 1.6 Hz, H-7), 9.01 (d, 1H, *J* = 1.9 Hz, H-3), 9.10 (d, 1H, *J* = 1.9 Hz, H-2), 9.21 (dd, 1H, *J* = 4.3, 1.6 Hz, H-9); ^13^C NMR (75 MHz, CDCl_3_) δ 123.6, 128.1, 128.4, 130.1, 136.1, 141.3, 144.4, 144.5, 145.5, 145.7, 150.7; anal. calcd for C_11_H_7_N_3_: C, 72.92; H, 3.89; N, 23.19; found: C, 72.97; H, 3.90; N, 23.17.

**9-Chloro-[1,2,5]selenadiazolo[3,4-*f*]quinoline (10).** A mixture of selenadiazolo[3,4-*f*]quinolone **9** (2.0 g, 8.0 mmol) and POCl_3_ (4 mL, 6.56 g, 42.8 mmol) was stirred at 90 °C for 3 h. After the reaction was complete, the mixture was cooled to 0 °C in an ice bath followed by the addition of crushed ice (~30 g) in one portion under stirring. Once the ice was melted, the resulting brown solution was stirred for 45 min and subsequently alkalised with a 20% NaOH solution under cooling in the ice bath. The grey–brown precipitate was collected by suction, washed with water and dried. Purification by FLC (silica gel, CHCl_3_, *R*_f_ = 0.21) afforded 9-chloroselenadiazoloquinoline **10** (1.90 g, 89%) as a pale yellow solid; mp 225–226 °C. ^1^H NMR (300 MHz, TFA-*d*) δ 8.02 (d, 1H, *J* = 9.7 Hz, H-4), 8.06 (d, 1H, *J* = 6.3 Hz, H-8), 8.26 (d, 1H, *J* = 9.7 Hz, H-5), 8.69 (d, 1H, *J* = 6.1 Hz, H-7); ^13^C NMR (75 MHz, TFA-*d*) δ 125.2, 126.1, 129.9, 134.5, 143.3, 145.8, 155.2, 158.6, 159.7; anal. calcd for C_9_H_4_ClN_3_Se: C, 40.25; H, 1.50; N, 15.65; found: C, 40.21; H, 1.51; N, 15.67.

**4-Chloro-5,6-diaminoquinoline (11).** 9-Chloroselenadiazoloquinoline **10** (0.50 g, 1.86 mmol) was added in small portions to a stirred suspension of SnCl_2_·2H_2_O (1.68 g, 7.44 mmol) in concentrated HCl (15 mL) at room temperature and stirring was continued for 30 min. Next, the reaction mixture was diluted with water (40 mL), the insoluble material was removed by filtration under reduced pressure and the filter cake was washed with water (20 mL). The filtrate was subsequently alkalised with saturated Na_2_CO_3_ solution and the resulting yellow suspension was extracted with ethyl acetate (6 × 75 mL). In the first extraction, precipitation of the product occurred between the organic and water phases due to its low solubility in ethyl acetate. In this case, the water phase was separated to leave the precipitate in the organic phase in a separatory funnel. Methanol (15 mL) was added to this suspension to dissolve the precipitated product and the resulting yellow solution was put aside. The water phase was extracted with 5 additional portions of ethyl acetate. The combined organic phases were dried with Na_2_SO_4_, filtered and evaporated under reduced pressure to afford 4-chlorodiaminoquinoline **11** (0.28 g, 77%) as a yellow–brown solid which was characterised without further purification; mp > 150 °C (dec.). ^1^H NMR (300 MHz, DMSO-*d*_6_) δ 5.13 (br s, 2H, NH_2_), 5.23 (br s, 2H, NH_2_), 7.27 (s, 2H, H-7, H-8), 7.28 (d, 1H, *J* = 4.5 Hz, H-3), 8.29 (d, 1H, *J* = 4.5 Hz, H-2); ^13^C NMR (75 MHz, DMSO-*d_6_*) δ 114.8, 118.9, 121.5, 121.6, 125.8, 132.8, 136.8, 144.52, 144.53; anal. calcd for C_9_H_8_ClN_3_: C, 55.83; H, 4.16; N, 21.70, found: C, 55.93; H, 4.12; N, 21.79.

## Supporting Information

File 1 ^1^H and ^13^C NMR spectra of compounds **2–6, 8, 10** and **11**.
