# Supplementary material for: New syntheses of 5,6- and 7,8-diaminoquinolines
Source: Beilstein J Org Chem. 2013 Nov 27;9:2669–74. doi: 10.3762/bjoc.9.302 (PMC3869217; doi:10.3762/bjoc.9.302)
Supplement: File 1 — 1H and 13C NMR spectra of compounds 2–6, 8, 10 and 11. [file Beilstein_J_Org_Chem-09-2669-s001.pdf]

Supporting Information

for

New syntheses of 5,6- and 7,8-diaminoquinolines

Maroš Bella[1]* and Viktor Milata[2]

Address: [1]Institute of Chemistry, Slovak Academy of Sciences, Dúbravská cesta 9, SK-845 38 Bratislava, Slovakia and [2]Department of Organic Chemistry, Faculty of Chemical and Food Technology, Slovak University of Technology, Radlinského 9, SK-812 37 Bratislava, Slovakia

Email: Maroš Bella – maros.bella@savba.sk

* Corresponding author

^{1}H and ^{13}C NMR spectra of compounds 2-6, 8, 10 and 11

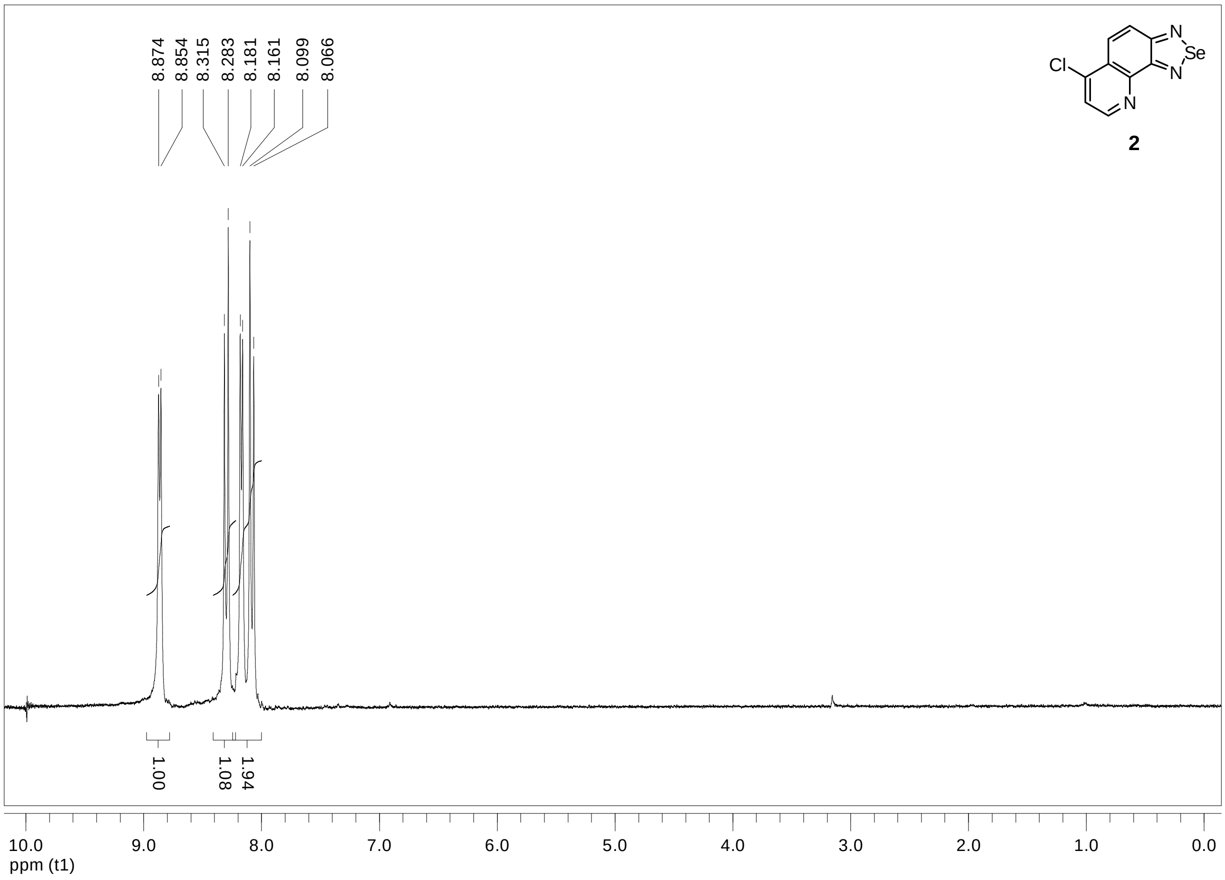
8.874
8.854
8.315
8.283
8.181
8.161
8.099
8.066
Cl
N
Se
N
N
2
1.00
1.08
1.94
10.0
9.0
8.0
7.0
6.0
5.0
4.0
3.0
2.0
1.0
0.0
ppm (t1)

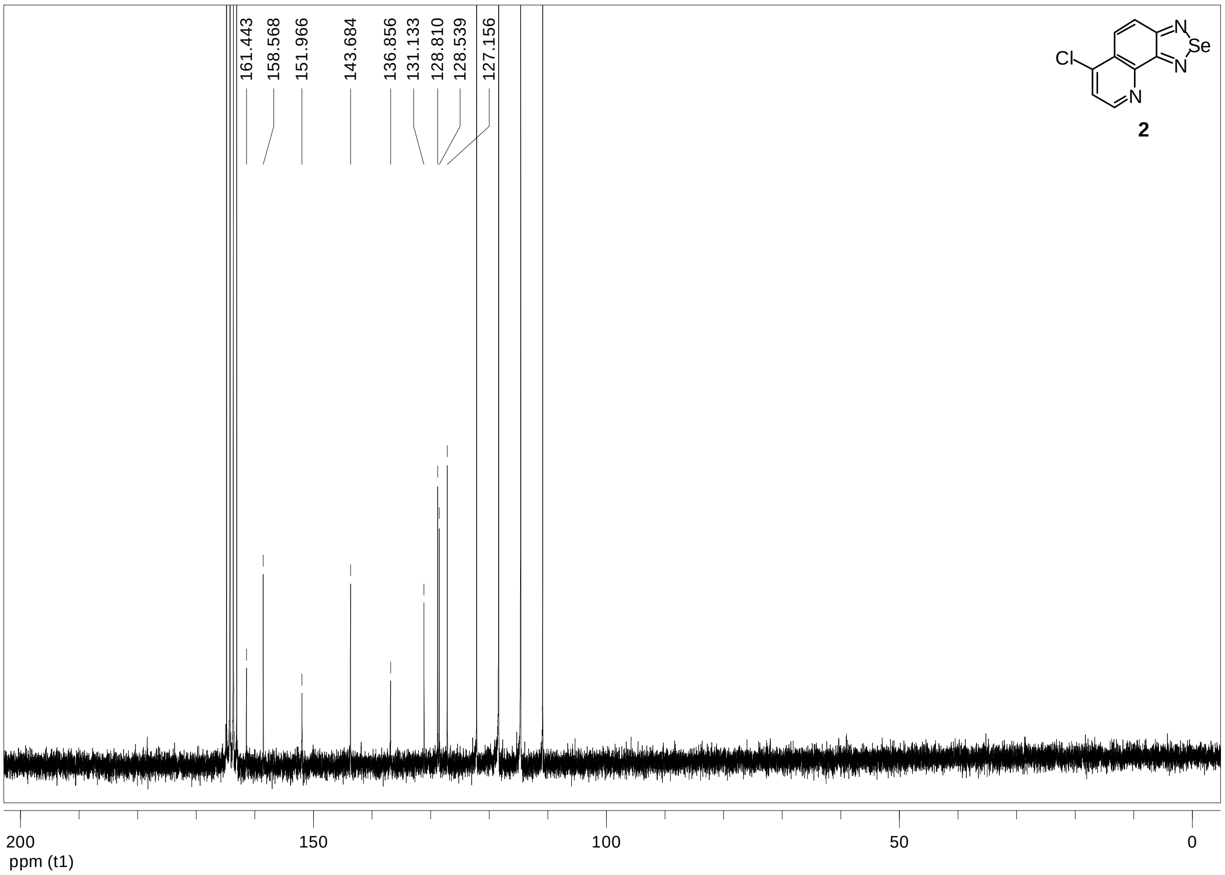
161.443
158.568
151.966
143.684
136.856
131.133
128.810
128.539
127.156
Cl
N
Se
N
N
2
200
150
100
50
0
ppm (t1)

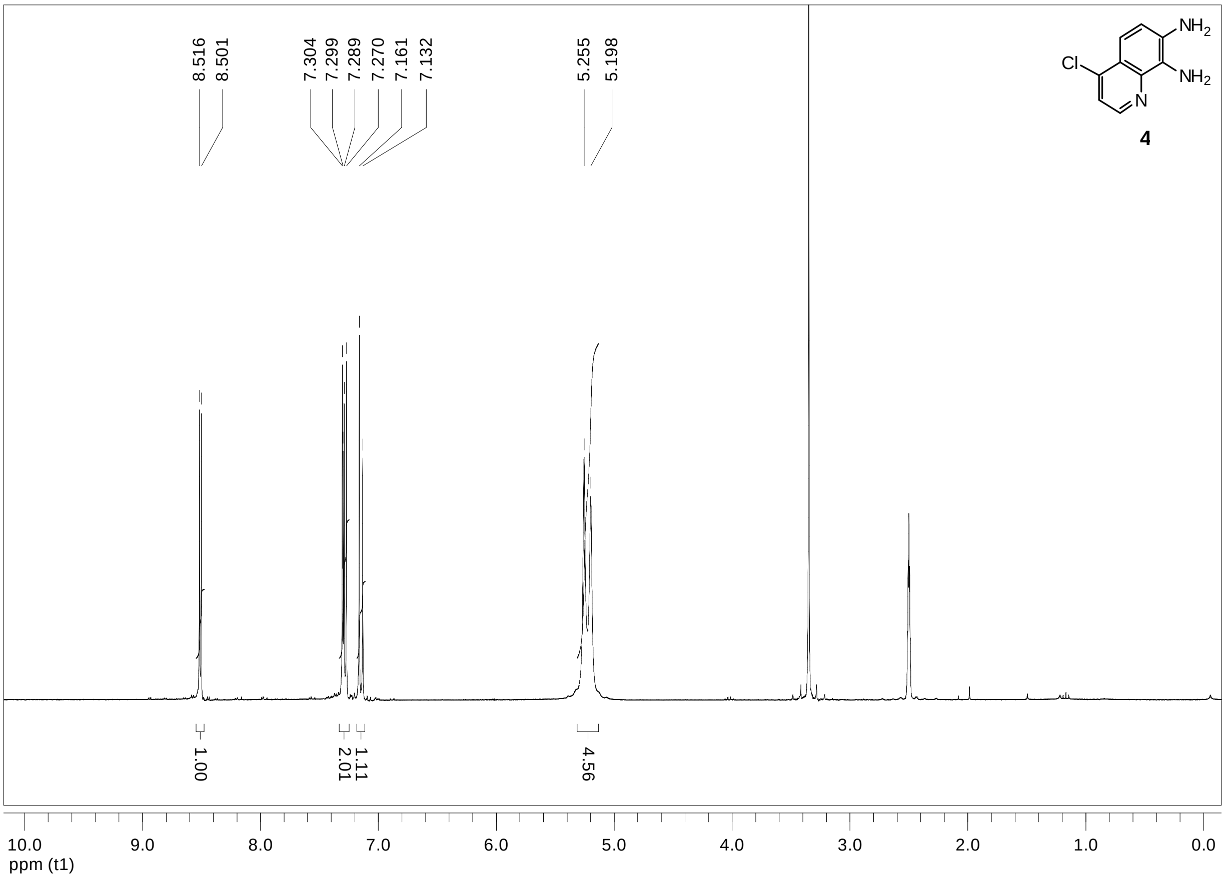
8.516
8.501
7.304
7.299
7.289
7.270
7.161
7.132
5.255
5.198
NH2
Cl
NH2
N
4
1.00
2.01
1.11
4.56
10.0
9.0
8.0
7.0
6.0
5.0
4.0
3.0
2.0
1.0
0.0
ppm (t1)

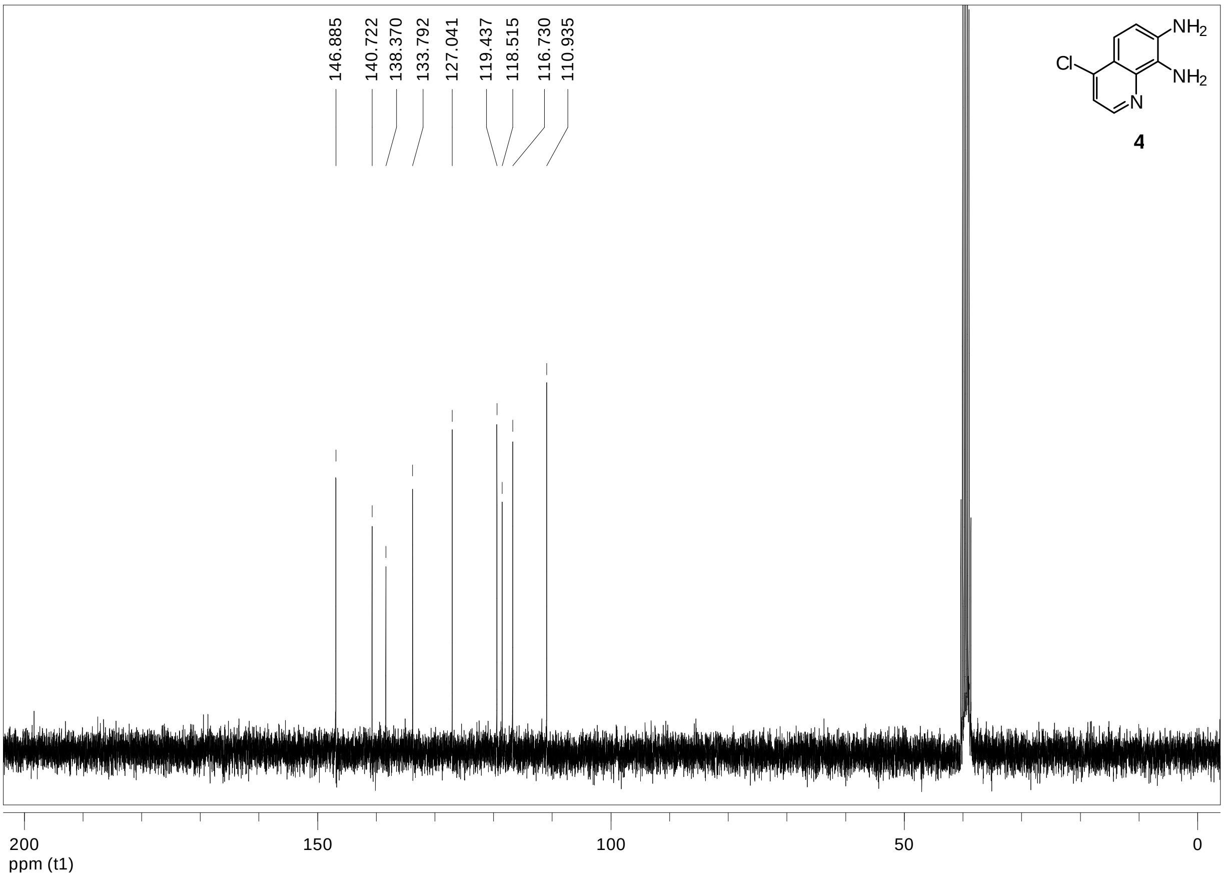
146.885
140.722
138.370
133.792
127.041
119.437
118.515
116.730
110.935
NH2
Cl
NH2
N
4
200
150
100
50
0
ppm (t1)

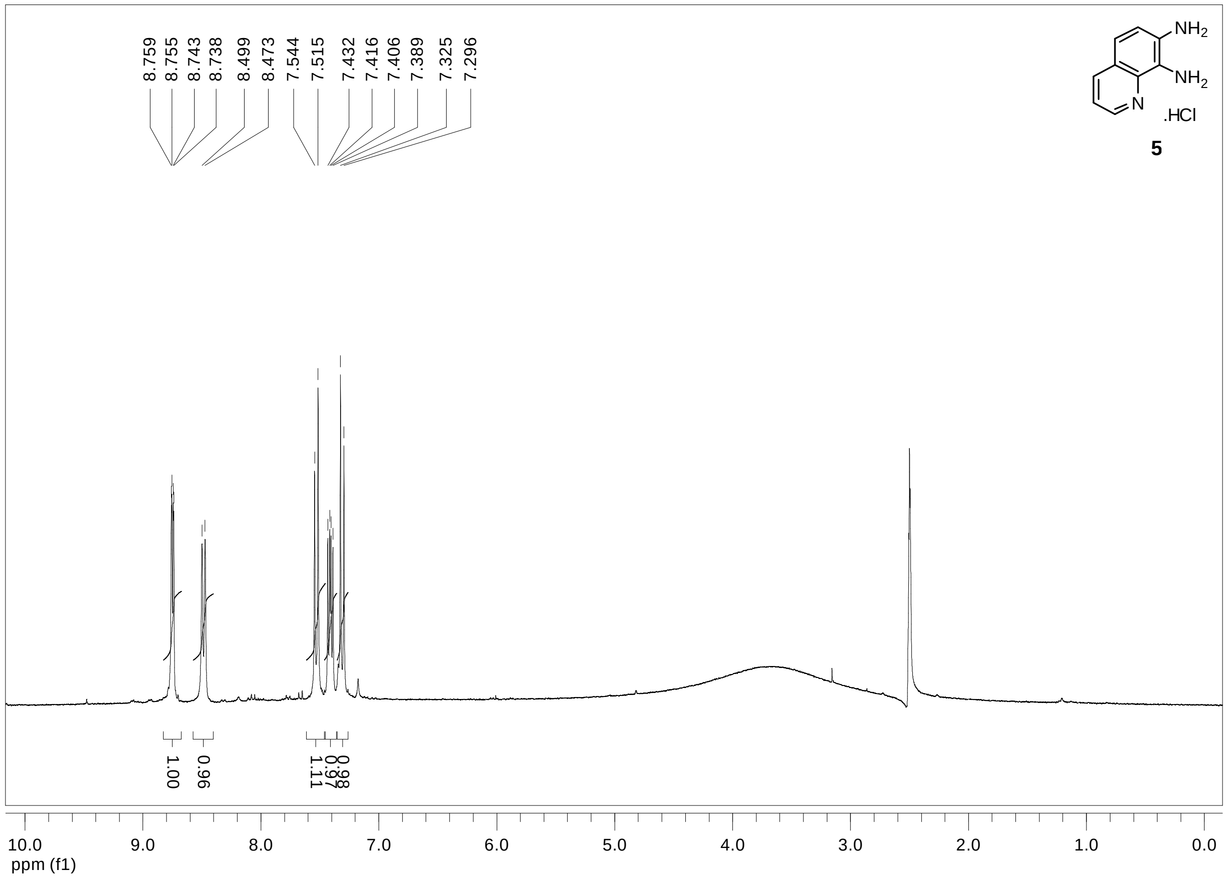
8.759
8.755
8.743
8.738
8.499
8.473
7.544
7.515
7.432
7.416
7.406
7.389
7.325
7.296
NH_2
NH_2
N
.HCl
5
1.00
0.96
1.11
0.97
0.98
10.0
9.0
8.0
7.0
6.0
5.0
4.0
3.0
2.0
1.0
0.0
ppm (f1)

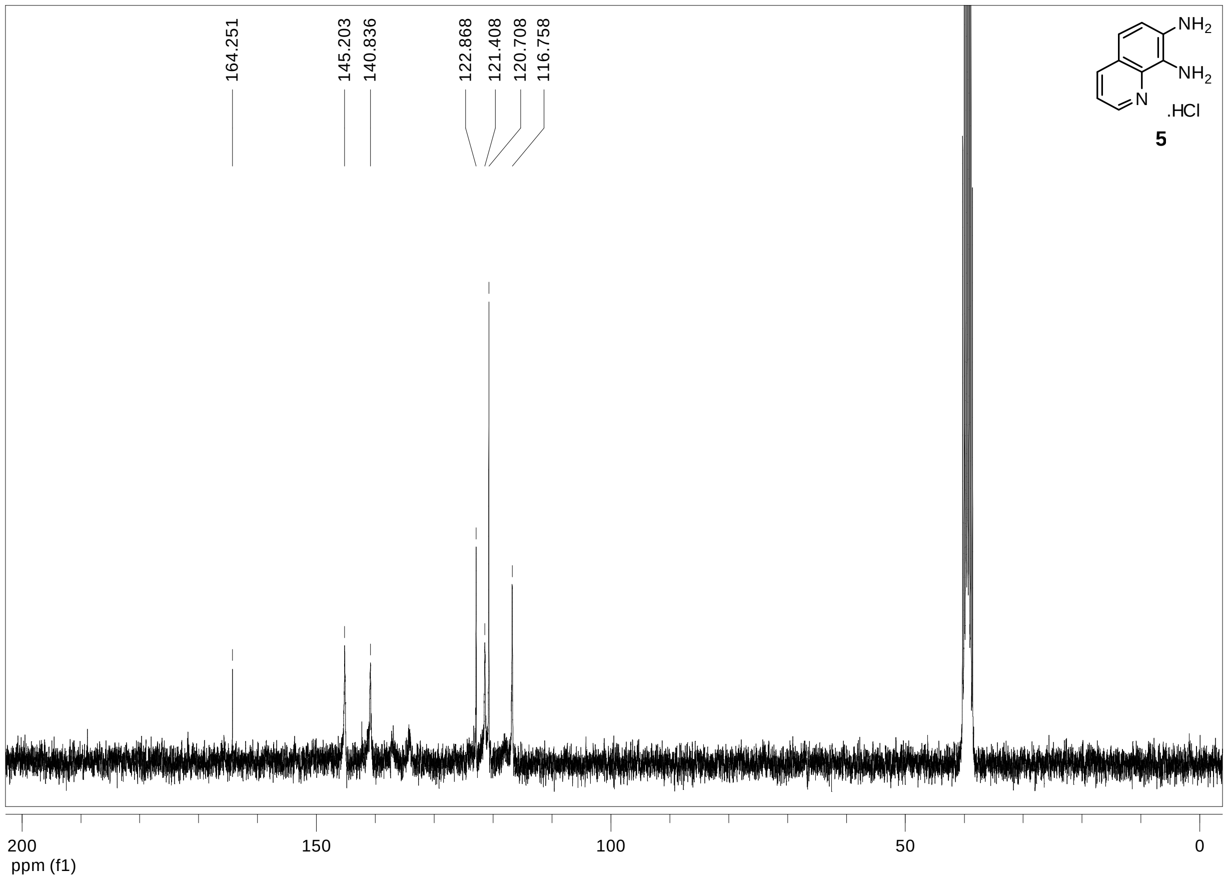
164.251
145.203
140.836
122.868
121.408
120.708
116.758
NH_2
NH_2
N
.HCl
5
200
150
100
50
0
ppm (f1)

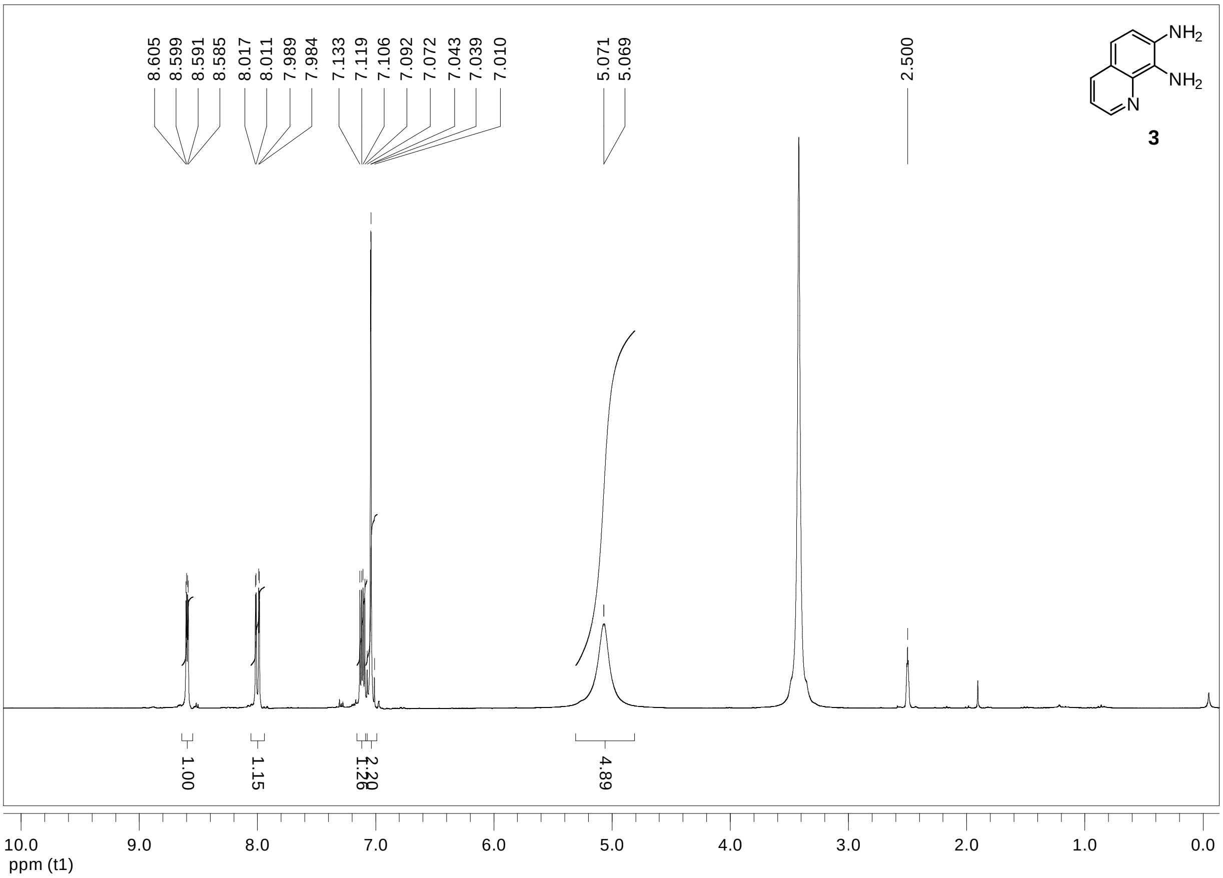
8.605
8.599
8.591
8.585
8.017
8.011
7.989
7.984
7.133
7.119
7.106
7.092
7.072
7.043
7.039
7.010
5.071
5.069
2.500
NH2
NH2
N
3
1.00
1.15
1.20
2.20
4.89
10.0
9.0
8.0
7.0
6.0
5.0
4.0
3.0
2.0
1.0
0.0
ppm (t1)

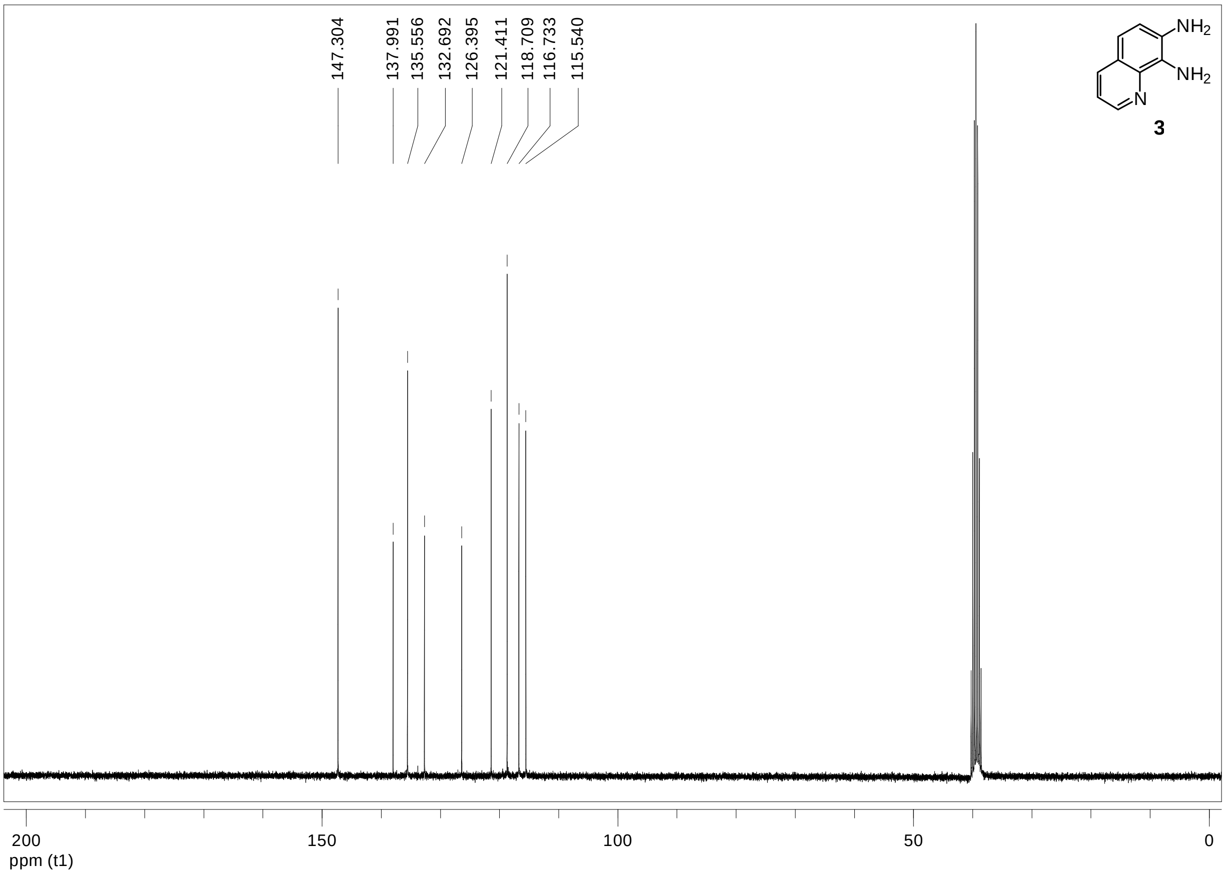
147.304
137.991
135.556
132.692
126.395
121.411
118.709
116.733
115.540
NH2
NH2
N
3
200
150
100
50
0
ppm (t1)

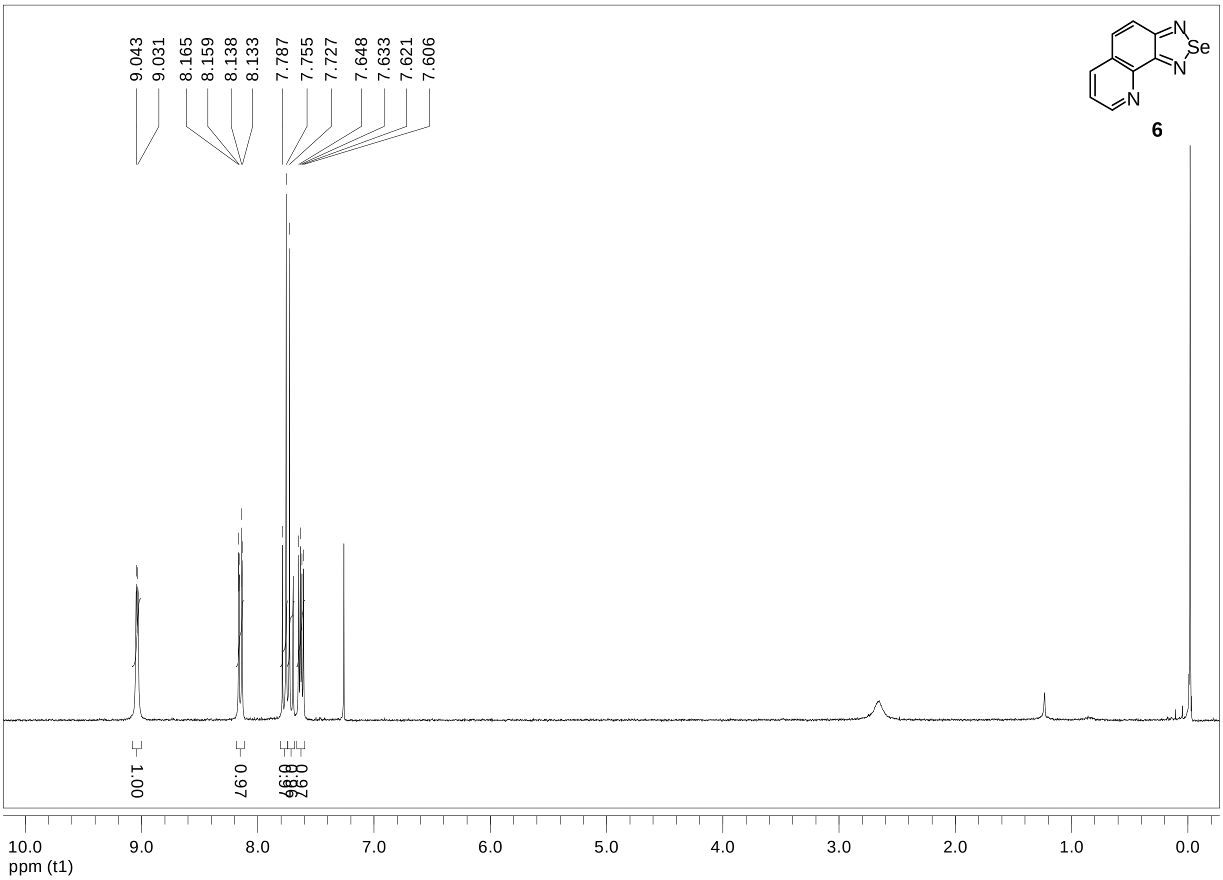
9.043
9.031
8.165
8.159
8.138
8.133
7.787
7.755
7.727
7.648
7.633
7.621
7.606
N
Se
N
N
6
1.00
0.97
10.0
9.0
8.0
7.0
6.0
5.0
4.0
3.0
2.0
1.0
0.0
ppm (t1)

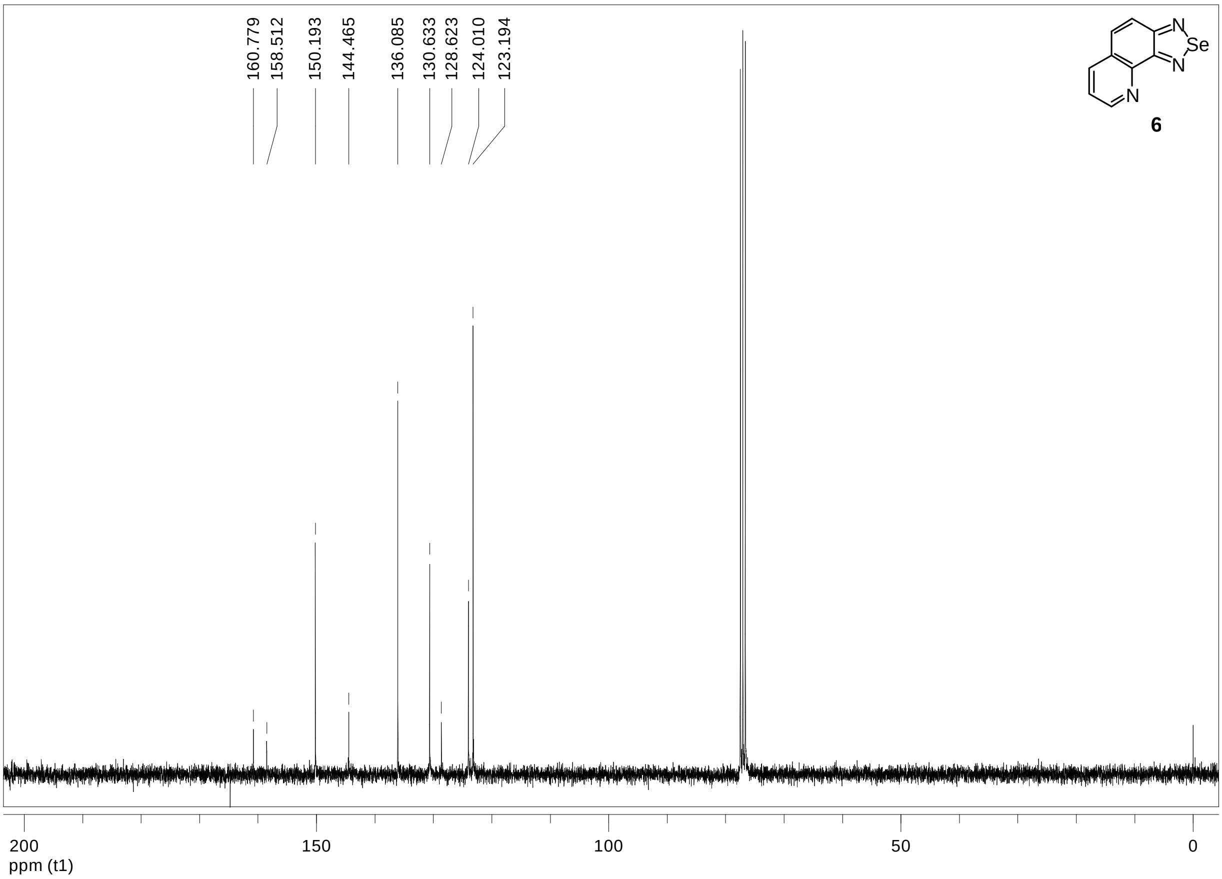
160.779
158.512
150.193
144.465
136.085
130.633
128.623
124.010
123.194
N
Se
N
N
6
200
150
100
50
0
ppm (t1)

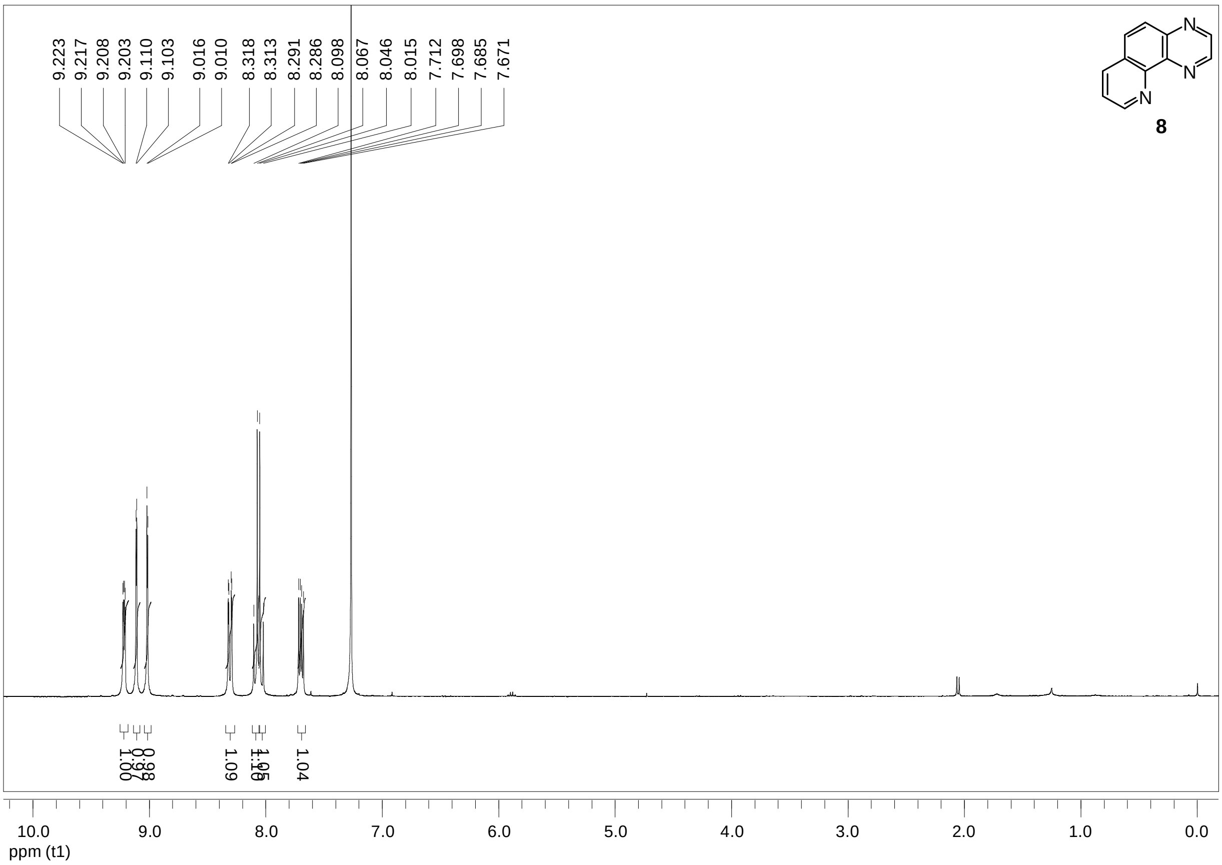
9.223
9.217
9.208
9.203
9.110
9.103
9.016
9.010
8.318
8.313
8.291
8.286
8.098
8.067
8.046
8.015
7.712
7.698
7.685
7.671
N
N
N
8
1.09
1.04
10.0
9.0
8.0
7.0
6.0
5.0
4.0
3.0
2.0
1.0
0.0
ppm (t1)

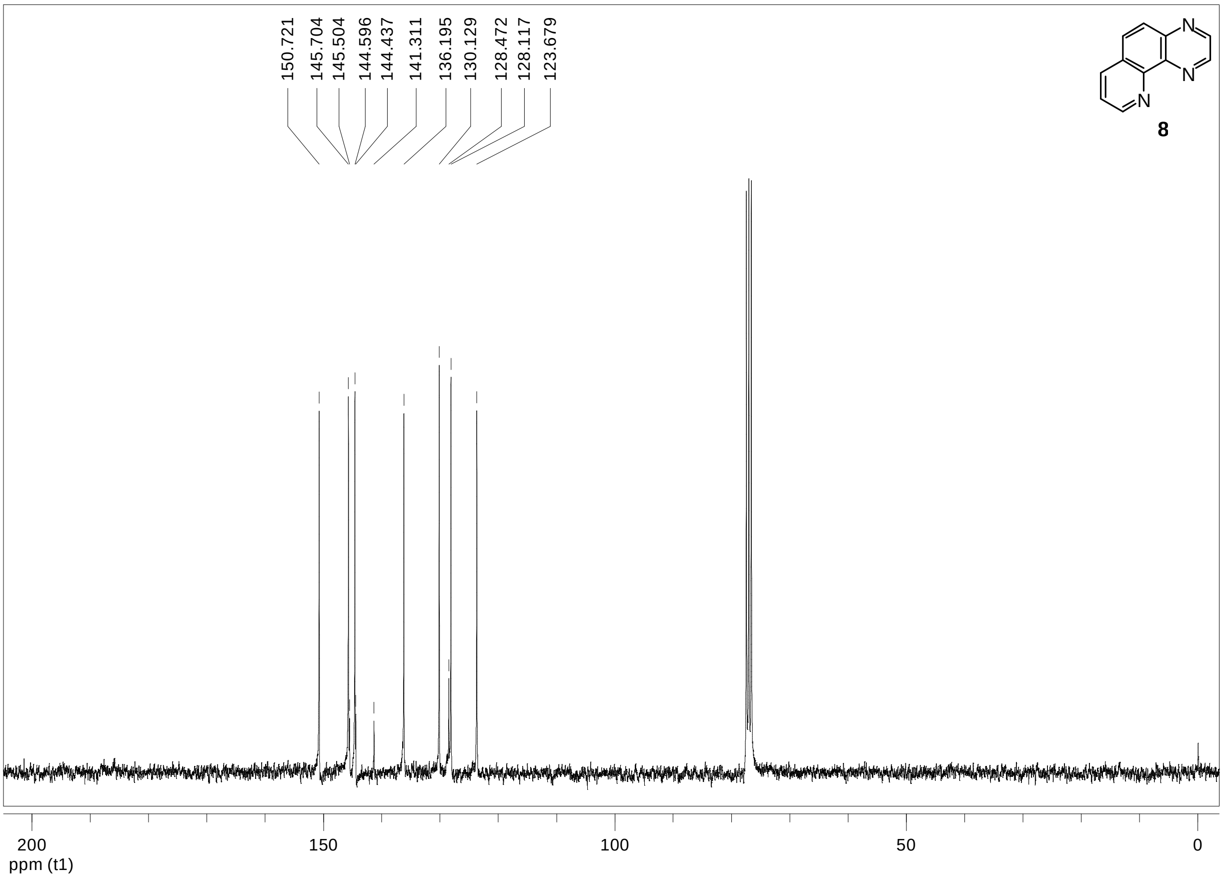
150.721
145.704
145.504
144.596
144.437
141.311
136.195
130.129
128.472
128.117
123.679
N
N
N
8
200
150
100
50
0
ppm (t1)

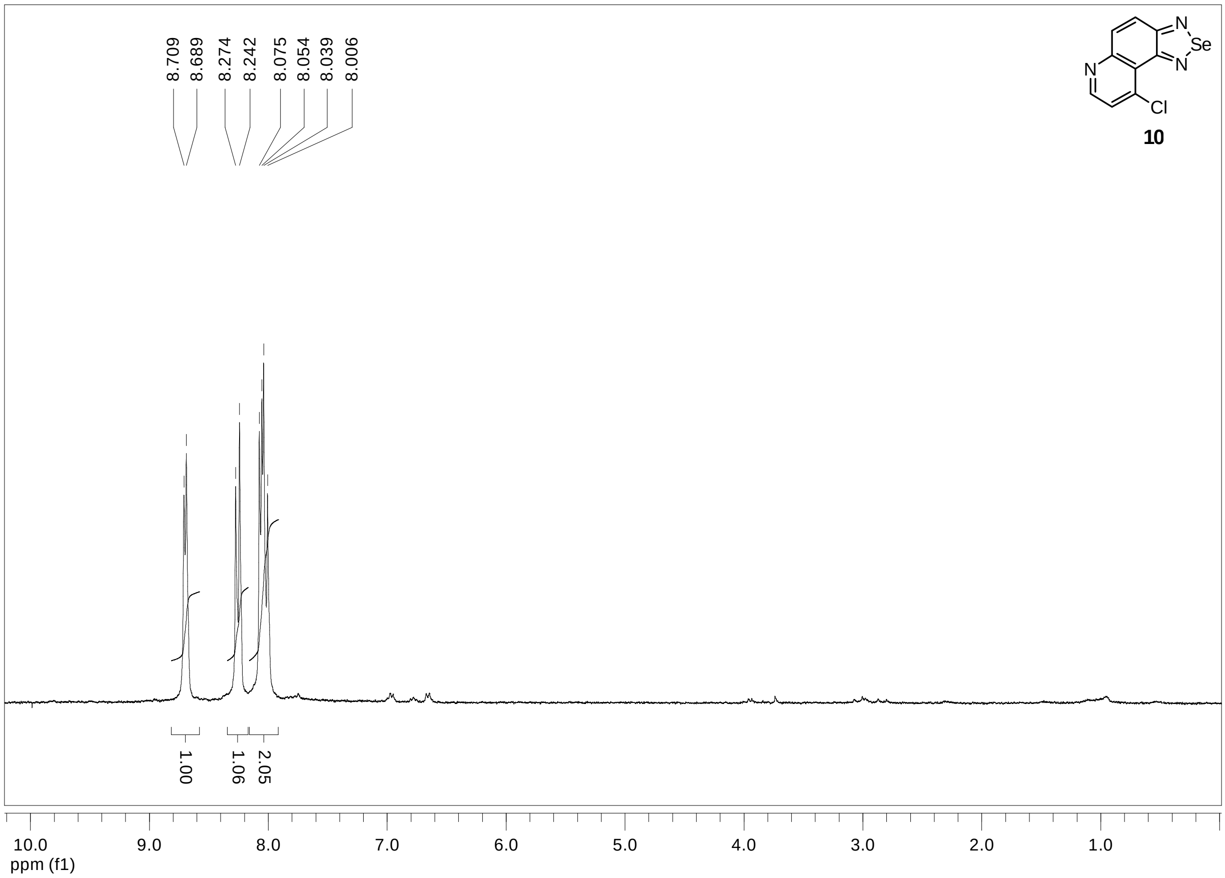
8.709
8.689
8.274
8.242
8.075
8.054
8.039
8.006
N
Se
N
N
Cl
10
1.00
1.06
2.05
10.0
9.0
8.0
7.0
6.0
5.0
4.0
3.0
2.0
1.0
ppm (f1)

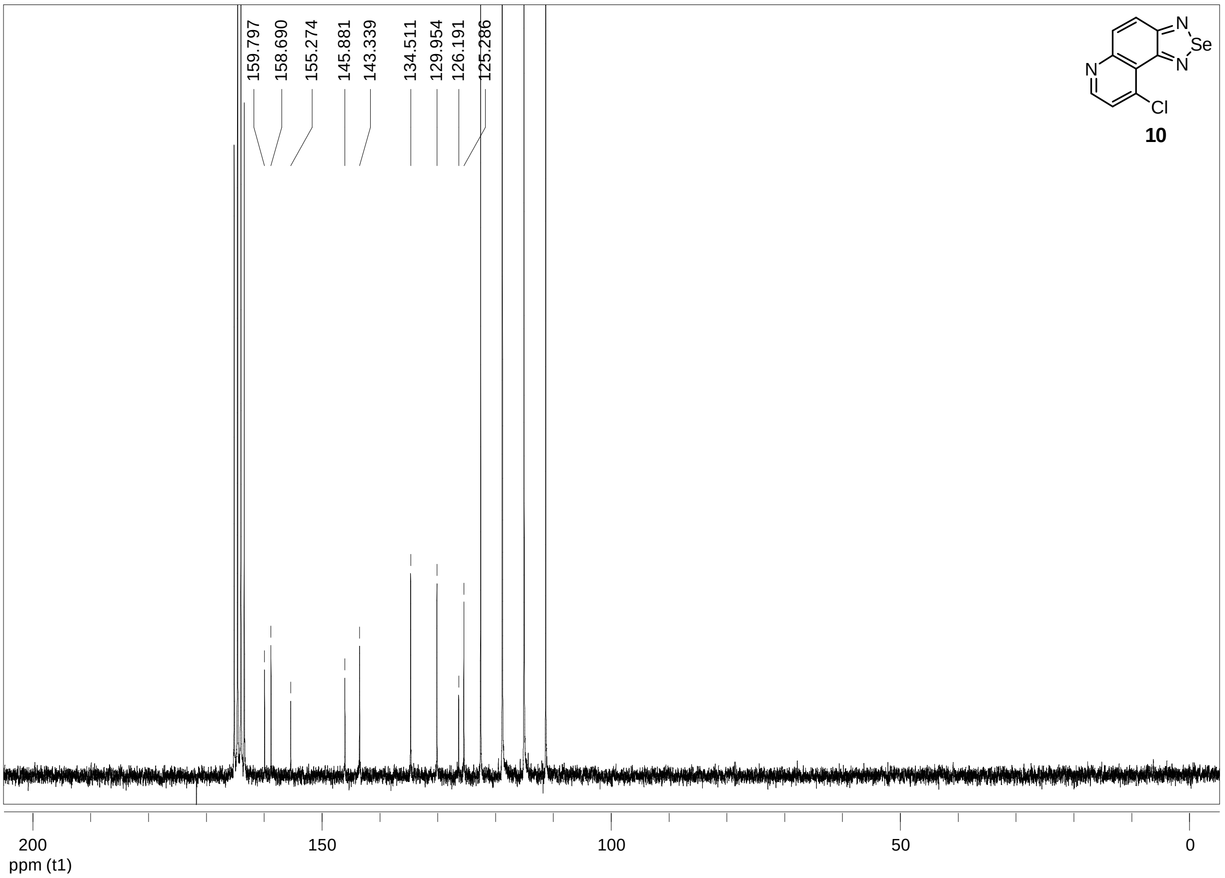
159.797
158.690
155.274
145.881
143.339
134.511
129.954
126.191
125.286
N
Se
N
N
Cl
10
200
150
100
50
0
ppm (t1)

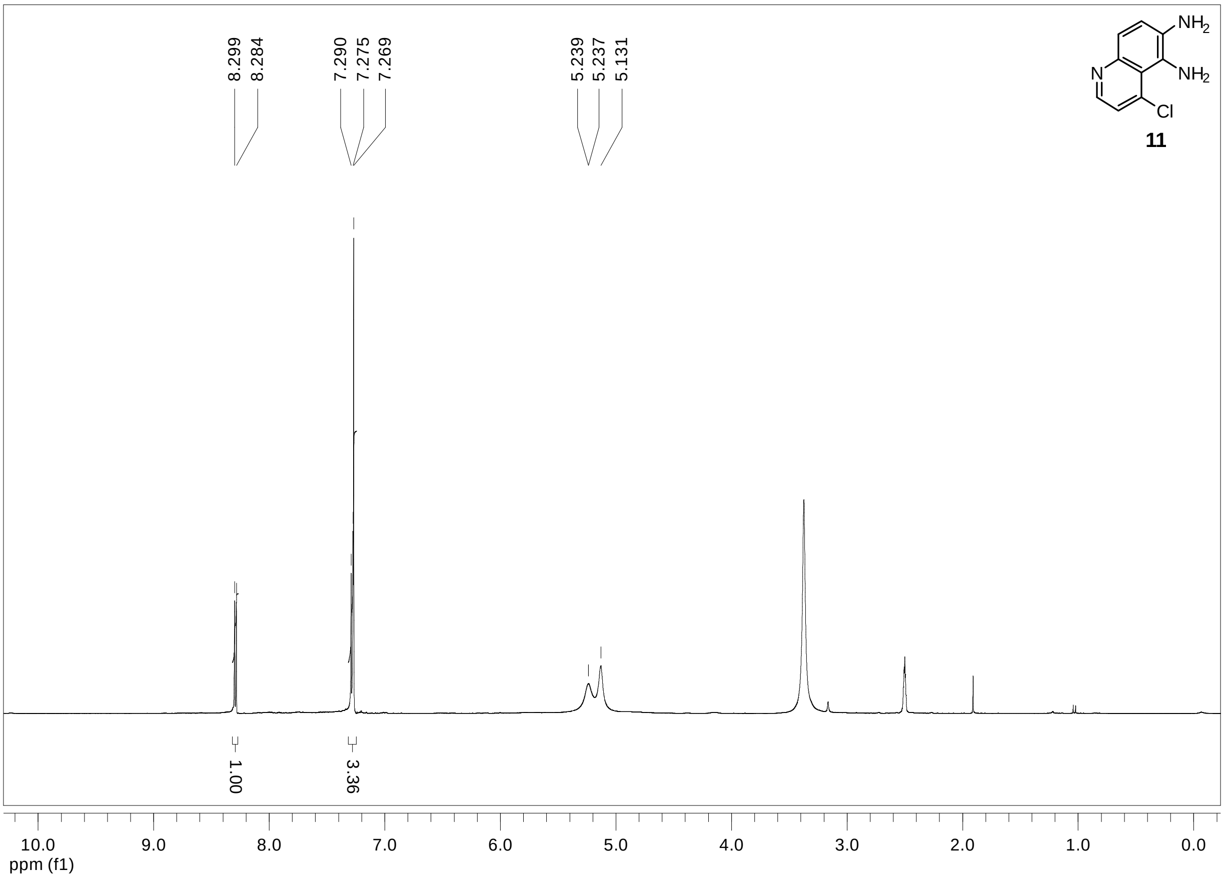
8.299
8.284
7.290
7.275
7.269
5.239
5.237
5.131
NH2
N
NH2
Cl
11
1.00
3.36
10.0
9.0
8.0
7.0
6.0
5.0
4.0
3.0
2.0
1.0
0.0
ppm (f1)

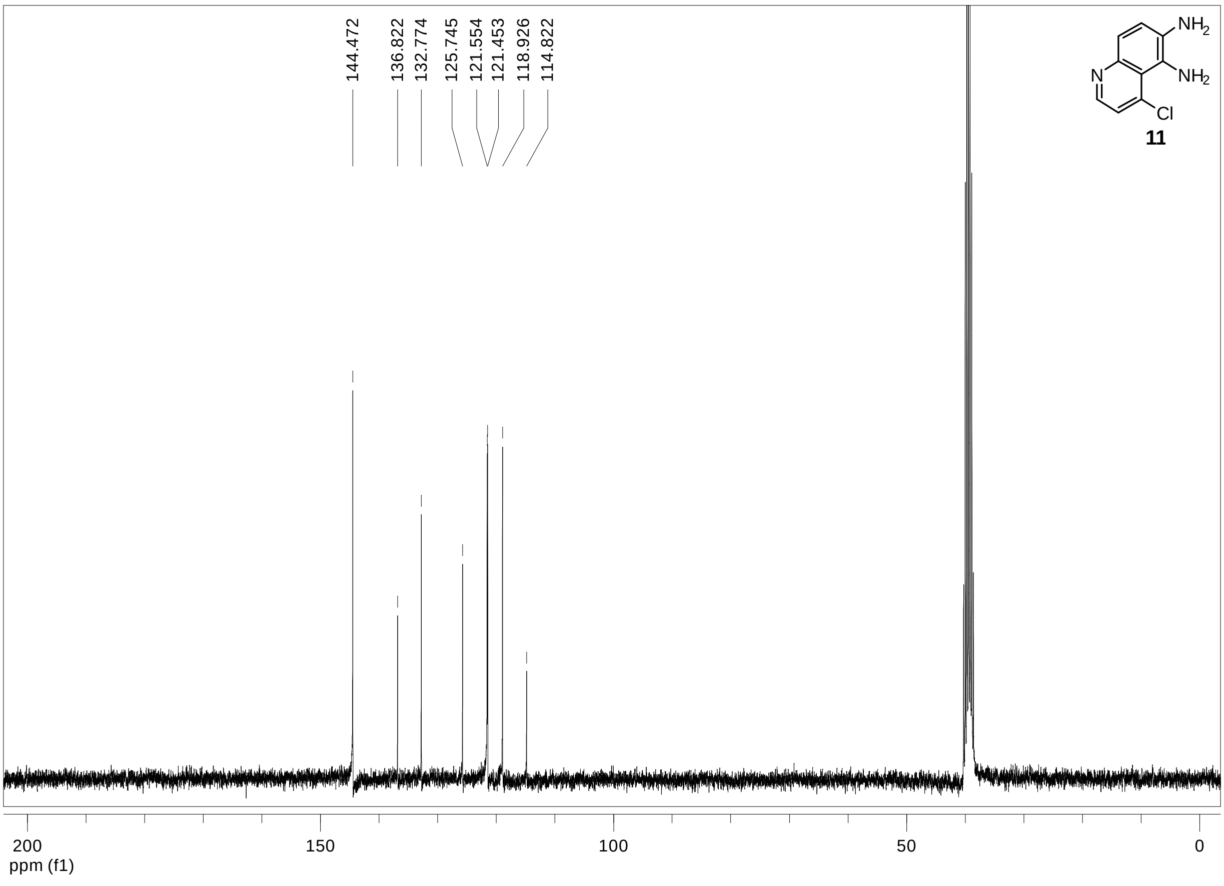
144.472
136.822
132.774
125.745
121.554
121.453
118.926
114.822
NH2
N
NH2
Cl
11
200
150
100
50
0
ppm (f1)
